# Supplementary figures and images for: qc3C: Reference-free quality control for Hi-C sequencing data
Source: PLoS Comput Biol. 2021 Oct 11;17(10):e1008839. doi: 10.1371/journal.pcbi.1008839 (PMC8530316; doi:10.1371/journal.pcbi.1008839)

**A**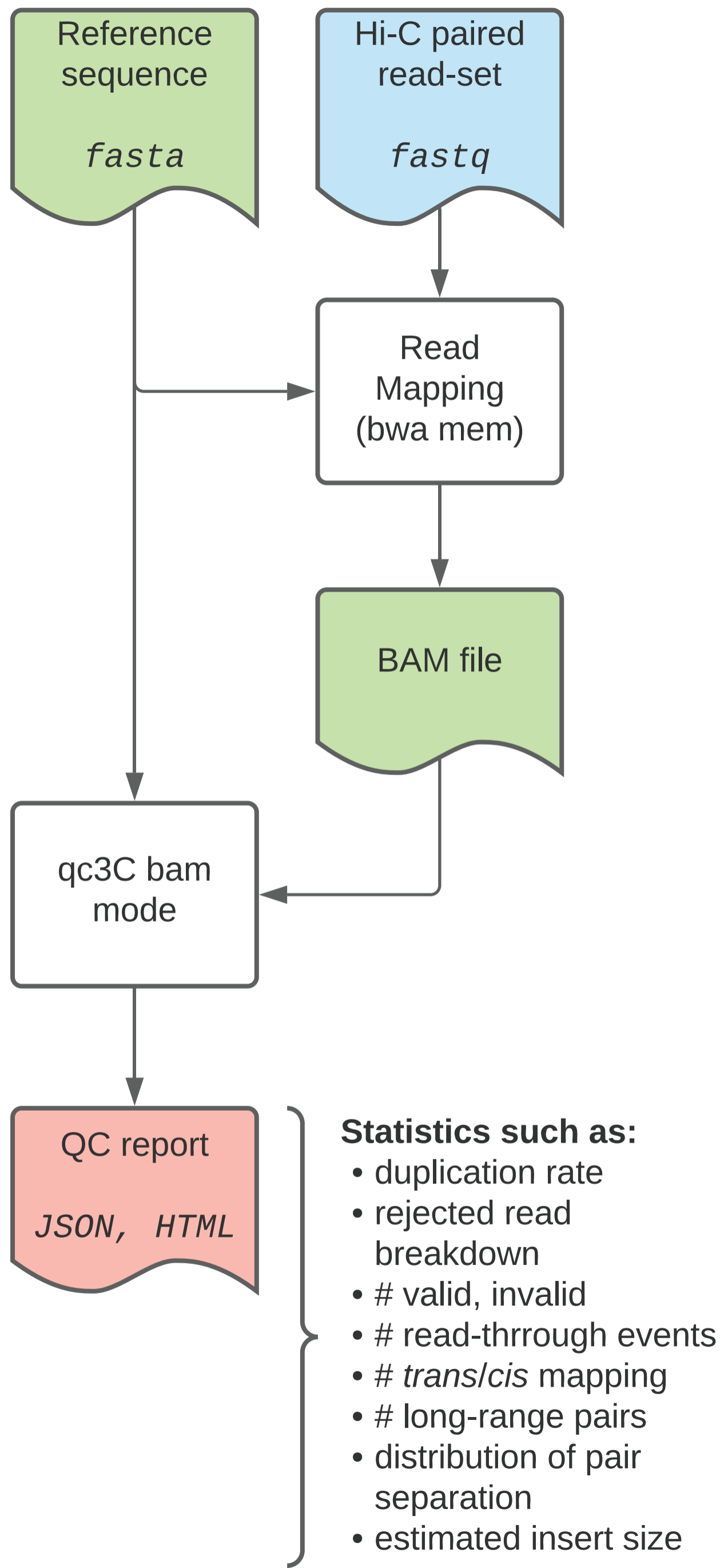**B**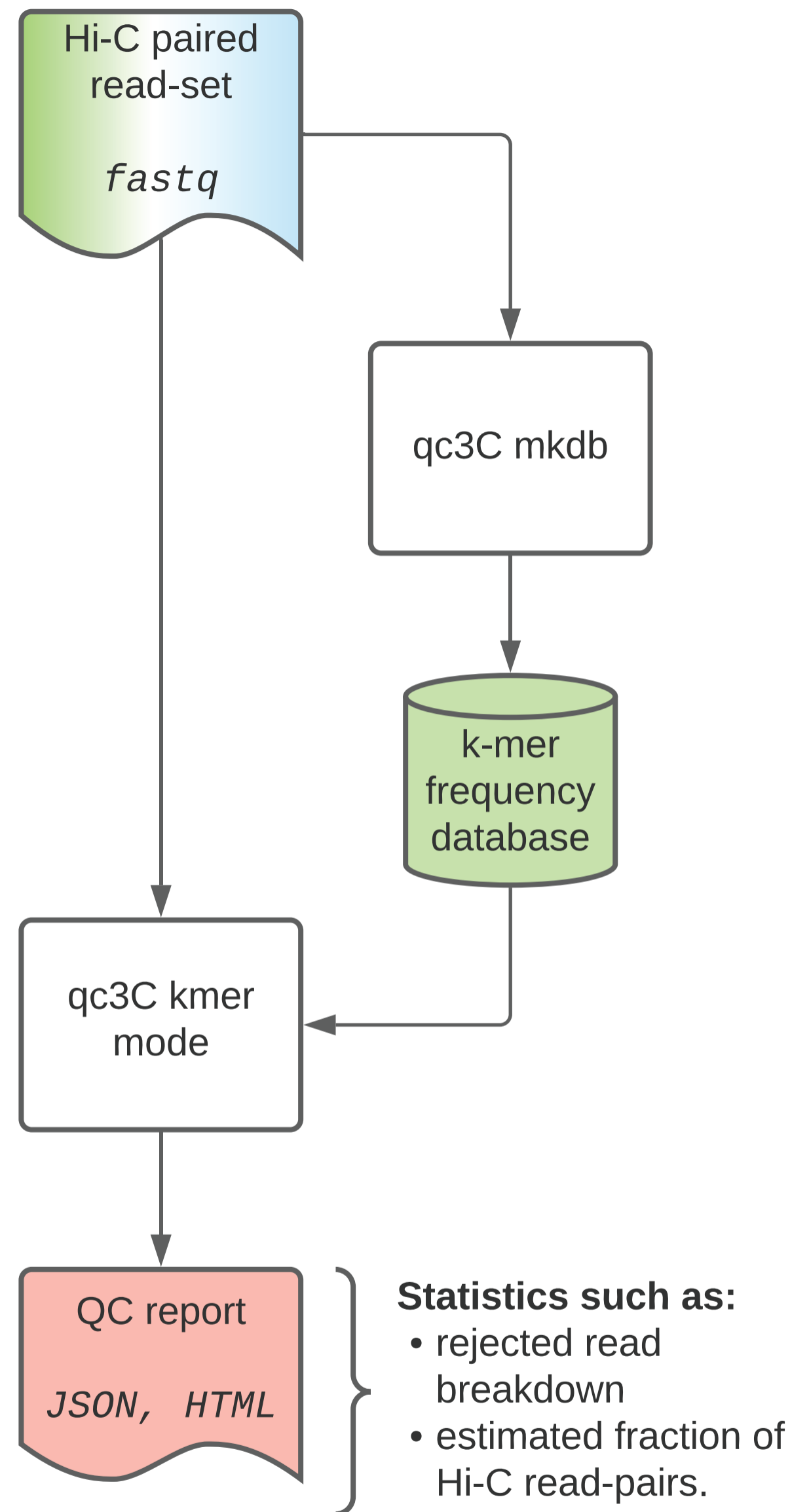

Supplement: S1 Fig — The two primary modes of operation A) BAM (reference-based) and B) KMER (reference-free) are invoked by the sub-commands qc3C bam and qc3C kmer. Additionally, the sub-command qc3C mkdb is provided as a convenience for constructing Jellyfish k-mer libraries. Reference-based BAM mode requires as input the reference in FASTA format and a user-made BAM file containing alignments of a Hi-C paired-end read-set to the supplied reference. Reference-free KMER mode requires as input only a Hi-C paired-end read-set in FASTQ format. If no k-mer database is supplied to KMER mode, qc3C will offer to create one, otherwise users can themselves create a database using the qc3C mkdb sub-command. The primary modes emit different QC reports, which is written to the console, and JSON and HTML formatted files. (PDF) [file pcbi.1008839.s001.pdf]

A

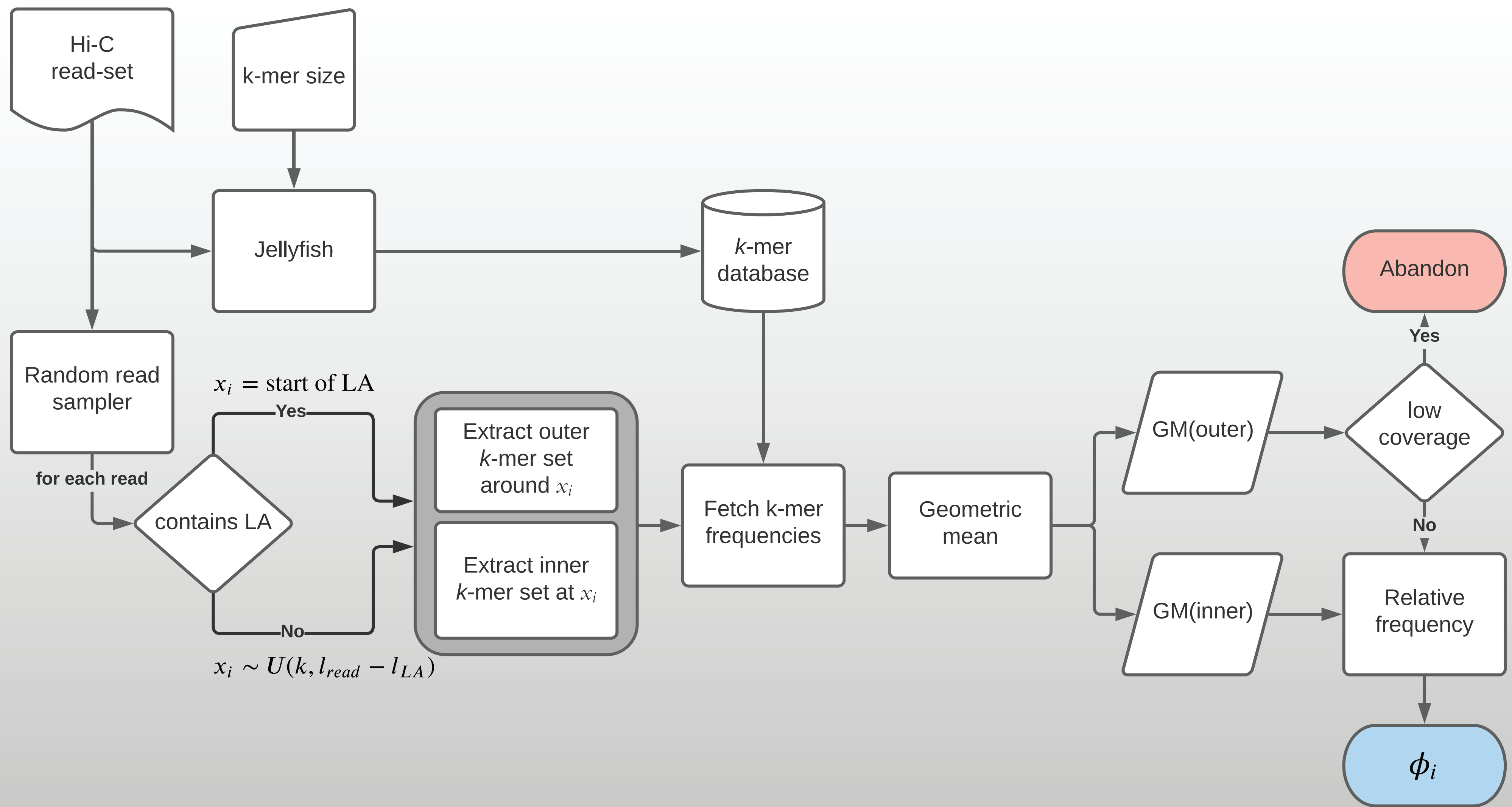

B

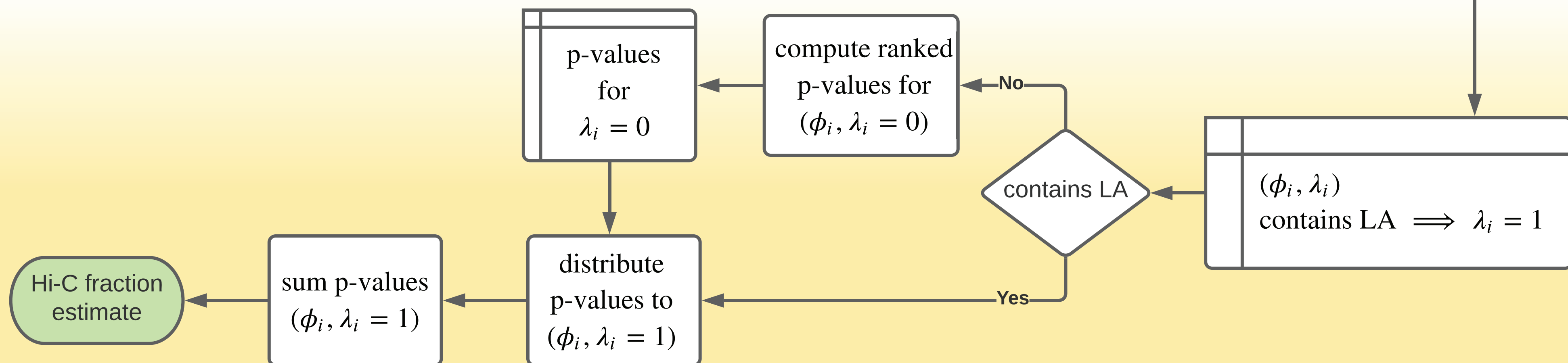

Supplement: S2 Fig — Symbolic workflow of the qc3C algorithm. A) Beginning with a Hi-C read-set, Jellyfish is used to generate a k-mer database for a user-selectable size k. Afterwards, observations of relative coverage ϕi are collected both around ligation artefacts (LA) and randomly for ordinary regions. B) Collected observations of ϕi for ordinary regions are used to compute empirical ranked p-values. Computed p-values are subsequently distributed to the observations of ϕi around LA and a final estimation of the fraction of Hi-C read-pairs is obtained as the weighted sum of these p-values. Bootstrapping is used to infer a 95% confidence interval. (PDF) [file pcbi.1008839.s002.pdf]

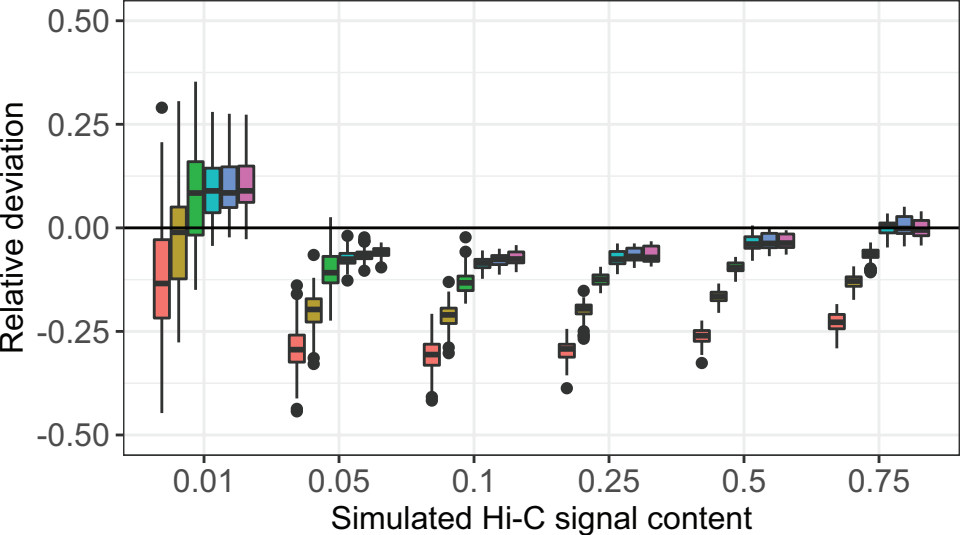

Supplement: S3 Fig — The relative deviation of predicted from actual Hi-C signal content using qc3C’s reference-free method when overlapping read-pairs are merged prior to analysis. The sweep has been partitioned so that bins contain 140 sample points of varying insert length (equivalent to Fig 4). For merged read-pairs, there is a small improvement in performance relative to unmerged read-pairs, however prediction at the lowest simulated Hi-C signal level (signal = 0.01) remains the least accurate. (PDF) [file pcbi.1008839.s003.pdf]

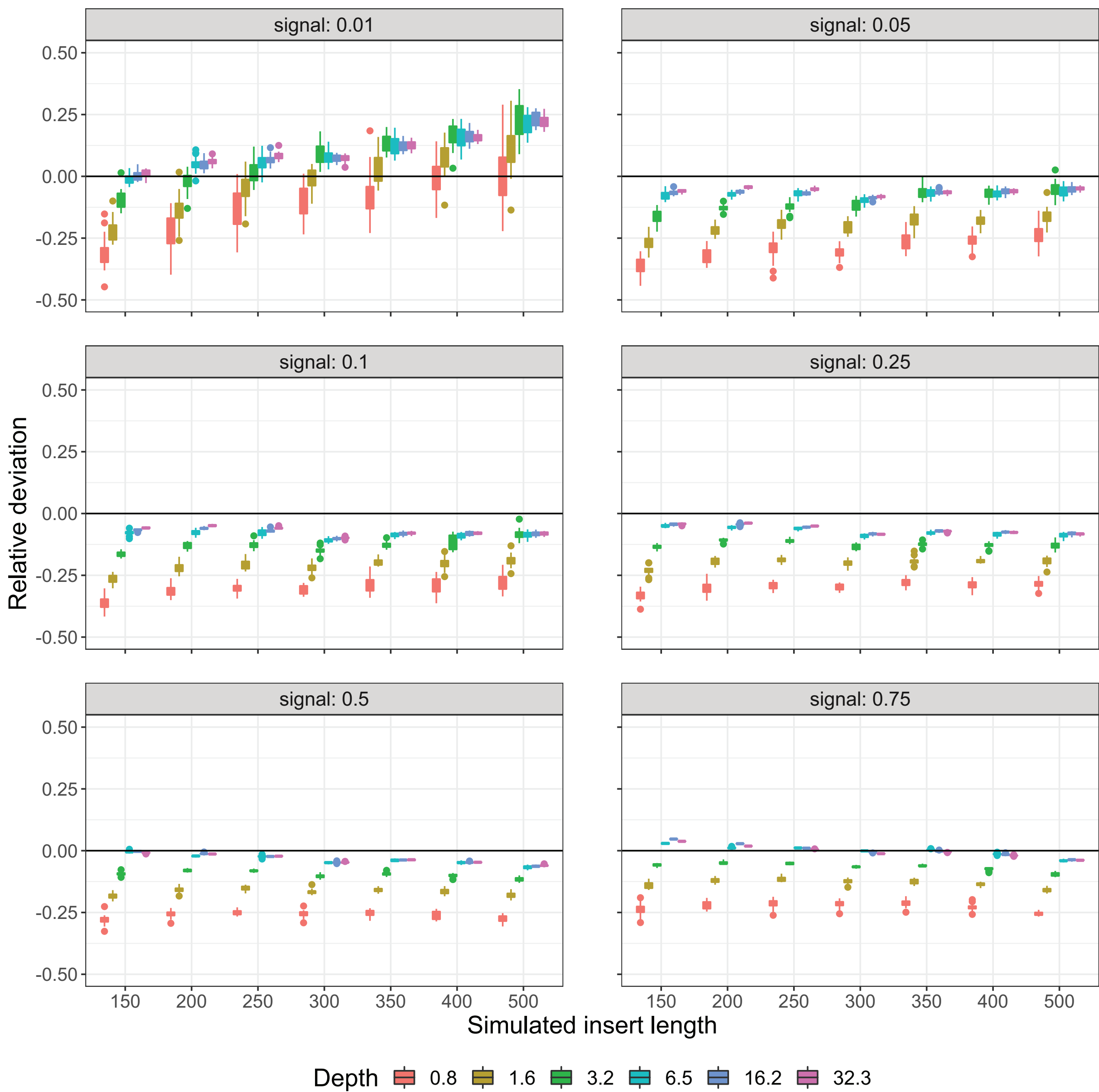

Supplement: S4 Fig — The sweep has been fully partitioned and all bins contain 20 replicates, differing in random seed only (equivalent to Figs 7 and 8). Relative deviation of predicted from actual Hi-C signal content closely resembles that of unmerged read-pairs. Since reads were simulated at a length of 150 bp, read-pair overlap is most pronounced when insert length was ≤ 300 bp. At insert lengths larger than this threshold, predictions become increasingly similar to the unmerged results. Overall, between merged and unmerged read-pairs, there are only slight differences in the mean and variation of replicates at equivalent points in the sweep. (PDF) [file pcbi.1008839.s004.pdf]
