# Supplementary material for: qc3C: Reference-free quality control for Hi-C sequencing data
Source: PLoS Comput Biol. 2021 Oct 11;17(10):e1008839. doi: 10.1371/journal.pcbi.1008839 (PMC8530316; doi:10.1371/journal.pcbi.1008839)
Supplement: S1 Supporting Information — Aside from reporting quality results to the user via the console, an analysis run produces a quality report written to disk in both HTML and JSON formats. The create if either output format can be disabled. The JSON format files can be imported by MultiQC. This zip archive includes example results of both BAM and KMER modes, as well as the resulting MultiQC report. (ZIP) [file pcbi.1008839.s005.zip › example_output/multiqc_report.html]

MultiQC Report


# Toggle navigation v1.10.dev0

Loading report..

- qc3C
  - BAM mode analysis details
  - BAM mode read parsing
  - BAM mode HiC-Pro validation
  - BAM mode long-range pairs
  - BAM mode distribution of fragment separation
  - BAM mode junction breakdown
  - K-mer mode runtime details
  - K-mer mode Hi-C fraction
  - K-mer mode read parsing
  - K-mer mode junction breakdown

Toolbox

### MultiQC Toolbox

#### Apply Highlight Samples

+

Regex mode off
help
 Clear

#### Apply Rename Samples

+

Click here for bulk input.

Paste two columns of a tab-delimited table here (eg. from Excel).

First column should be the old name, second column the new name.

Add

Regex mode off
help
 Clear

#### Apply Show / Hide Samples

Hide matching samples

Show only matching samples

+

Regex mode off
help
 Clear

#### Export Plots

- Images
- Data

px

px

Aspect ratio

PNG
JPEG
SVG

Plot scaling

X

Download the raw data used to create the plots in this report below:

Format:

Tab-separated
Comma-separated
JSON

Note that additional data was saved in `multiqc_data` when this report was generated.

---

##### Choose Plots

 All
 None

---


   Download Plot Images

If you use plots from MultiQC in a publication or presentation, please cite:

> **MultiQC: Summarize analysis results for multiple tools and samples in a single report**  
> *Philip Ewels, Måns Magnusson, Sverker Lundin and Max Käller*  
> Bioinformatics (2016)  
> doi: 10.1093/bioinformatics/btw354  
> PMID: 27312411

#### Save Settings

You can save the toolbox settings for this report to the browser.

 Save


---

#### Load Settings

Choose a saved report profile from the dropdown box below:

[ select ]

Load
 Delete
 Set default
 Clear default

#### About MultiQC

This report was generated using MultiQC, version 1.10.dev0

You can see a YouTube video describing how to use MultiQC reports here:
https://youtu.be/qPbIlO\_KWN0

For more information about MultiQC, including other videos and
extensive documentation, please visit http://multiqc.info

You can report bugs, suggest improvements and find the source code for MultiQC on GitHub:
https://github.com/ewels/MultiQC

MultiQC is published in Bioinformatics:

> **MultiQC: Summarize analysis results for multiple tools and samples in a single report**  
> *Philip Ewels, Måns Magnusson, Sverker Lundin and Max Käller*  
> Bioinformatics (2016)  
> doi: 10.1093/bioinformatics/btw354  
> PMID: 27312411

# 

A modular tool to aggregate results from bioinformatics analyses across many samples into a single report.

#### JavaScript Disabled

MultiQC reports use JavaScript for plots and toolbox functions. It looks like
you have JavaScript disabled in your web browser. Please note that many of the report
functions will not work as intended.

Loading report..

Report
generated on 2021-02-10, 17:03
based on data in:
`/Users/cerebis/git/qc3C/Feb7_2020/drr`

---

×
don't show again

**Welcome!** Not sure where to start?  
Watch a tutorial video
  *(6:06)*

## qc3C

qc3C provides reference-free and BAM based quality control for Hi-C data

### BAM mode analysis details

This table details various alignment features which are potentially of interest to
researchers attempting to assess the quality of a Hi-C library.

Copy table

 Configure Columns

 Sort by highlight

 Plot
Showing 17/17 rows and 6/8 columns.

| Sample | Date | Run Mode | Digest | Accepted pairs | Read length | Insert length | Unobserved | Read-thru |
| --- | --- | --- | --- | --- | --- | --- | --- | --- |
| DRR177157 | 2021-02-10 | bam | HindIII | 200000 | 127bp | 217bp | 0.0% | 35.8% |
| DRR177158 | 2021-02-10 | bam | HindIII | 200000 | 127bp | 233bp | 0.0% | 39.1% |
| DRR177159 | 2021-02-10 | bam | DpnII | 200000 | 127bp | 219bp | 0.0% | 49.1% |
| DRR177160 | 2021-02-10 | bam | DpnII | 200000 | 127bp | 218bp | 0.0% | 46.0% |
| DRR177161 | 2021-02-10 | bam | Sau3AI MluCI | 200000 | 127bp | 511bp | 53.2% | 5.1% |
| DRR177162 | 2021-02-10 | bam | Sau3AI MluCI | 200000 | 127bp | 550bp | 56.6% | 3.6% |
| DRR177163 | 2021-02-10 | bam | DpnII HinfI | 200000 | 127bp | 297bp | 19.3% | 30.8% |
| DRR177164 | 2021-02-10 | bam | DpnII HinfI | 200000 | 127bp | 319bp | 24.4% | 29.9% |
| DRR177165 | 2021-02-10 | bam | HindIII | 200000 | 127bp | 248bp | 4.1% | 41.4% |
| DRR177166 | 2021-02-10 | bam | HindIII | 200000 | 127bp | 248bp | 4.1% | 36.7% |
| DRR177167 | 2021-02-10 | bam | HindIII | 200000 | 151bp | 219bp | 0.0% | 42.3% |
| DRR177168 | 2021-02-10 | bam | HindIII | 200000 | 151bp | 227bp | 0.0% | 46.0% |
| DRR177169 | 2021-02-10 | bam | DpnII | 200000 | 151bp | 218bp | 0.0% | 57.7% |
| DRR177170 | 2021-02-10 | bam | DpnII | 200000 | 151bp | 213bp | 0.0% | 53.7% |
| DRR177171 | 2021-02-10 | bam | Sau3AI MluCI | 200000 | 151bp | 485bp | 41.2% | 5.2% |
| DRR177172 | 2021-02-10 | bam | DpnII HinfI | 200000 | 151bp | 314bp | 8.0% | 37.1% |
| DRR177173 | 2021-02-10 | bam | HindIII | 200000 | 151bp | 237bp | 0.0% | 49.6% |

×

#### Qc3C Bam Runtime Table: Columns

Uncheck the tick box to hide columns. Click and drag the handle on the left to change order.

Show All
Show None

| Sort | Visible | Group | Column | Description | ID | Scale |
| --- | --- | --- | --- | --- | --- | --- |
| || |  | qc3C | Date | Analysis time stamp | `b_run_timestamp` | None |
| || |  | qc3C | Run Mode | Analysis mode used | `b_mode` | None |
| || |  | qc3C | Digest | Enzymes used in digest | `b_enzymes` | None |
| || |  | qc3C | Accepted pairs | Number of pairs accepted for analysis | `b_n_accepted_pairs` | None |
| || |  | qc3C | Read length | Average observed read length | `b_mean_readlen` | None |
| || |  | qc3C | Insert length | Inferred insert size | `b_obs_insert_mean` | None |
| || |  | qc3C | Unobserved | Fraction of total insert extent that was unobservable | `b_unobs_fraction` | None |
| || |  | qc3C | Read-thru | Fraction of reads whose alignments end in a cutsite and whose sequence continues for the full junction | `b_p_read_thru` | None |

Close

---

### BAM mode read parsing

This figure displays a breakdown of proportion of parsed reads rejected due to various
criteria and the proportion that were accepted.

Number of Reads
Percentages

loading..

---

### BAM mode HiC-Pro validation Help

A visualisation of the read-pair categories devised by
HiC-Pro.

As the field has moved from 6-cutter to 4-cutter enzymes, and subsequently dual-enzyme digests, the
higher density of sites has made this framework less useful, since it has become increasingly easy
to satisfy the intervening site criteria.

Number of Reads
Percentages

loading..

---

### BAM mode long-range pairs Help

This plot visualises the breakdown of read-pairs based on separation distance.

The breakdown of separation distance is only calculated for *cis*-mapping pairs.

Ideally, Hi-C proximity ligation should produce many pairs which are greater than 1000 bp apart.
However, these statistics are strongly influenced by the state of the reference. For draft assemblies
the distance at which pairs can map is limited by the degree of fragmentation and length of contigs.
As a result, many more pairs will be categorised as *trans*-mapping and pairs which are truly
inter-molecular cannot be distinguished from those which are merely inter-contig.

Number of Reads
Percentages

loading..

---

### BAM mode distribution of fragment separation Help

This figure displays the a normalised histogram of read-pair separation binned uniformly in
log-space.

Due to the binning strategy, the x-axis is log-scaled and visually accommodates pair separations up
to 1 million bp. The inferred insert size for each library is represented by a dashed, grey vertical
line. The y-axis is log-scaled by default, allowing the density attributed to long-range pairs to be
more easily seen.

A characteristic of Hi-C libraries, is the presence of a large peak below 1000 bp. qc3C attributes
this to regular (and undesirable) shotgun pairs creeping through the Hi-C protocol. The peak is used
by qc3C to infer the insert size, which is later employed to estimate unobservable extent of
inserts.

**Note:** the inferred insert size can be significantly smaller than what a sequencing facility
might report the experimentally determined insert size to be. This discrepancy can be explained by
the failure to account for the additional adapter sequence when fragments are assessed during
library preparation.

Density
Log10 [Density]

loading..

---

### BAM mode junction breakdown Help

This figure displays the frequency at which a library's possible junction sequences
are actually observed in the reads. (*Trivial single-digests are ignored*)

For trivial single-enzyme digests, there is only one possible junction sequence and so the
result for these experiments are not plotted. For dual-enzyme (such as Phase Genomics) there are
four potential junctions, while for dual-enzyme digests with one ambiguous site (such as Arima
Genomics) there are 16 possible junction sequences.

How efficiently the more complicated library protocols are at producing hybrid junctions is
possibly just a point of interest.

Junctions are named for which enzymes was responsible for creating the 5' and 3' ends.
E.g. `Sau3AI/MluCI` would involve two different enzymes, while `Sau3AI/Sau3AI` only one, as
would be the case in a single-enzyme digest. Proceeding the name is the actual junction
sequence.

The junctions are grouped by their 5' and then 3' enzyme, while the color spectrum used across
each bar aims to emphasise these enzymatic sources.

**Note:** in BAM mode, the counts **are** controlled for false positives, in the sense that read
alignments must terminate at a cutsite, but the read sequence must continue and contain the
observed junction.

Counts
Percentages

loading..

---

### K-mer mode runtime details

This table includes user specified input options, observed read-length and unobservable fraction.

Copy table

 Configure Columns

 Sort by highlight

 Plot
Showing 17/17 rows and 5/8 columns.

| Sample | Date | Run Mode | k | Digest | Accepted reads | Insert length | Read length | Unobservable extent |
| --- | --- | --- | --- | --- | --- | --- | --- | --- |
| DRR177157 | 2021-02-10 | kmer | 24 | HindIII | 200000 | 214bp | 127bp | 3.1% |
| DRR177158 | 2021-02-10 | kmer | 24 | HindIII | 200000 | 231bp | 127bp | 3.6% |
| DRR177159 | 2021-02-10 | kmer | 24 | DpnII | 200000 | 216bp | 127bp | 4.7% |
| DRR177160 | 2021-02-10 | kmer | 24 | DpnII | 200000 | 215bp | 127bp | 4.5% |
| DRR177161 | 2021-02-10 | kmer | 24 | Sau3AI MluCI | 200000 | 509bp | 127bp | 54.4% |
| DRR177162 | 2021-02-10 | kmer | 24 | Sau3AI MluCI | 200000 | 549bp | 127bp | 57.9% |
| DRR177163 | 2021-02-10 | kmer | 24 | DpnII HinfI | 200000 | 295bp | 127bp | 22.8% |
| DRR177164 | 2021-02-10 | kmer | 24 | DpnII HinfI | 200000 | 315bp | 127bp | 27.7% |
| DRR177165 | 2021-02-10 | kmer | 24 | HindIII | 200000 | 244bp | 127bp | 7.9% |
| DRR177166 | 2021-02-10 | kmer | 24 | HindIII | 200000 | 244bp | 127bp | 10.0% |
| DRR177167 | 2021-02-10 | kmer | 24 | HindIII | 200000 | 216bp | 151bp | 1.6% |
| DRR177168 | 2021-02-10 | kmer | 24 | HindIII | 200000 | 225bp | 151bp | 2.0% |
| DRR177169 | 2021-02-10 | kmer | 24 | DpnII | 200000 | 215bp | 151bp | 2.5% |
| DRR177170 | 2021-02-10 | kmer | 24 | DpnII | 200000 | 210bp | 151bp | 2.2% |
| DRR177171 | 2021-02-10 | kmer | 24 | Sau3AI MluCI | 200000 | 486bp | 151bp | 41.8% |
| DRR177172 | 2021-02-10 | kmer | 24 | DpnII HinfI | 200000 | 311bp | 151bp | 11.2% |
| DRR177173 | 2021-02-10 | kmer | 24 | HindIII | 200000 | 234bp | 151bp | 2.3% |

×

#### Qc3C Kmer Runtime Table: Columns

Uncheck the tick box to hide columns. Click and drag the handle on the left to change order.

Show All
Show None

| Sort | Visible | Group | Column | Description | ID | Scale |
| --- | --- | --- | --- | --- | --- | --- |
| || |  | qc3C | Date | Analysis time stamp | `k_run_timestamp` | None |
| || |  | qc3C | Run Mode | Analysis mode used | `k_mode` | None |
| || |  | qc3C | k | Library k-mer size | `k_kmer_size` | None |
| || |  | qc3C | Digest | Enzymes used in digest | `k_enzymes` | None |
| || |  | qc3C | Accepted reads | Number of reads accepted for analysis | `k_n_accepted_reads` | None |
| || |  | qc3C | Insert length | User-specified insert size | `k_mean_insert` | None |
| || |  | qc3C | Read length | Observed average read length | `k_mean_readlen` | None |
| || |  | qc3C | Unobservable extent | Estimated mean of the unobservable portion of fragments | `k_unobs_fraction` | unobs\_mean |

Close

---

### K-mer mode Hi-C fraction Help

This table lists the inferred proportion of Hi-C proximity ligation fragments.

Here, **Mean adjusted Hi-C fraction** represents the best estimate of the proportion of a library's
read-pairs which are a product of proximity ligation. This figure is arrived at by correcting the
raw estimate for the fraction of insert extent which was not observable.

The observable extent is limited by the length of reads relative to the supplied insert size, as
well as a further constraint on flanking sequence around any suspected junction sequence.

Copy table

 Configure Columns

 Sort by highlight

 Plot
Showing 17/17 rows and 2/2 columns.

| Sample | Mean raw Hi-C fraction | Mean adjusted Hi-C fraction |
| --- | --- | --- |
| DRR177157 | 40.4% | 43.1% |
| DRR177158 | 45.5% | 49.0% |
| DRR177159 | 51.0% | 56.5% |
| DRR177160 | 49.3% | 54.4% |
| DRR177161 | 6.8% | 15.4% |
| DRR177162 | 4.8% | 11.8% |
| DRR177163 | 41.5% | 56.2% |
| DRR177164 | 41.0% | 59.4% |
| DRR177165 | 49.4% | 55.8% |
| DRR177166 | 43.8% | 51.9% |
| DRR177167 | 46.0% | 47.9% |
| DRR177168 | 51.3% | 53.8% |
| DRR177169 | 55.4% | 59.0% |
| DRR177170 | 52.7% | 55.8% |
| DRR177171 | 6.7% | 11.6% |
| DRR177172 | 47.1% | 54.9% |
| DRR177173 | 53.4% | 56.3% |

×

#### Qc3C Kmer Signal Table: Columns

Uncheck the tick box to hide columns. Click and drag the handle on the left to change order.

Show All
Show None

| Sort | Visible | Group | Column | Description | ID | Scale |
| --- | --- | --- | --- | --- | --- | --- |
| || |  | qc3C | Mean raw Hi-C fraction | Estimated mean of Hi-C fraction from only the observable extent | `k_raw_fraction` | None |
| || |  | qc3C | Mean adjusted Hi-C fraction | Estimated mean of Hi-C fraction adjusted for unobservable extent | `k_adj_fraction` | None |

Close

---

### K-mer mode read parsing

This figure displays a breakdown of proportion of parsed reads rejected due to various
criteria and the proportion that were accepted.

Number of Reads
Percentages

loading..

---

### K-mer mode junction breakdown Help

This figure displays the frequency at which a library's possible junction sequences
are actually observed in the reads. (*Trivial single-digests are ignored*)

```
For trivial single-enzyme digests, there is only one possible junction sequence and so the 
result for these experiments are not plotted. For dual-enzyme (such as Phase Genomics) there are 
four potential junctions, while for dual-enzyme digests with one ambiguous site (such as Arima 
Genomics) there are 16 possible junction sequences.

How efficiently the more complicated library protocols are at producing hybrid junctions is 
possibly just a point of interest.

Junctions are named for which enzymes was responsible for creating the 5' and 3' ends.
E.g. `Sau3AI/MluCI` would involve two different enzymes, while `Sau3AI/Sau3AI` only one, as 
would be the case in a single-enzyme digest. Proceeding the name is the actual junction 
sequence.

The junctions are grouped by their 5' and then 3' enzyme, while the color spectrum used across 
each bar aims to emphasise these enzymatic sources.
```

**Note:** in k-mer mode, the counts are not controlled for false positives.

Counts
Percentages

loading..

**MultiQC v1.10.dev0**
- Written by Phil Ewels,
available on GitHub.

This report uses HighCharts,
jQuery,
jQuery UI,
Bootstrap,
FileSaver.js and
clipboard.js.

×

### Plot Table Data

Select Column

Select Column

Please select two table columns.

Close

×

### Regex Help

Toolbox search strings can behave as regular expressions (regexes). Click a button below to see an example of it in action. Try modifying them yourself in the text box.

`^` (start of string)
`$` (end of string)
`[]` (character choice)
`\d` (shorthand for `[0-9]`)
`\w` (shorthand for `[0-9a-zA-Z_]`)
`.` (any character)
`\.` (literal full stop)
`()` `|` (group / separator)
`*` (prev char 0 or more)
`+` (prev char 1 or more)
`?` (prev char 0 or 1)
`{}` (char num times)
`{,}` (count range)

```
samp_1
samp_1_edited
samp_2
samp_2_edited
samp_3
samp_3_edited
prepended_samp_1
tmp_samp_1_edited
tmpp_samp_1_edited
tmppp_samp_1_edited
#samp_1_edited.tmp
samp_11
samp_11111
```

See regex101.com for a more heavy duty testing suite.

Close
