# Supplementary material for: qc3C: Reference-free quality control for Hi-C sequencing data
Source: PLoS Comput Biol. 2021 Oct 11;17(10):e1008839. doi: 10.1371/journal.pcbi.1008839 (PMC8530316; doi:10.1371/journal.pcbi.1008839)
Supplement: S1 Supporting Information — Aside from reporting quality results to the user via the console, an analysis run produces a quality report written to disk in both HTML and JSON formats. The create if either output format can be disabled. The JSON format files can be imported by MultiQC. This zip archive includes example results of both BAM and KMER modes, as well as the resulting MultiQC report. (ZIP) [file pcbi.1008839.s005.zip › example_output/bam_out/DRR177163/report.qc3C.html]

qc3C report


|  |  |
| --- | --- |
| mode | bam |
| runtime\_info | |  |  | | --- | --- | | qc3C\_version | qc3C 0.5rc10 | | run\_timestamp | 2021-02-10 05:00:36.288114 | |
| input\_args | |  |  | | --- | --- | | bam\_file | DRR177163.bam | | fasta\_file | GCF\_000230535.1\_PelSin\_1.0\_genomic.fna.gz | | enzymes | - DpnII - HinfI | | seed | 12345 | | sample\_rate | None | | min\_mapq | 60 | | max\_obs | 200000 | |
| n\_parsed\_reads | 819424 |
| n\_analysed\_reads | 811402 |
| n\_accepted\_reads | 503298 |
| n\_skipped\_reads | 0 |
| n\_unmapped | 8022 |
| n\_low\_mapq | 158515 |
| n\_ref\_len | 1386 |
| n\_secondary | 0 |
| n\_supplementary | 163238 |
| n\_duplicate | 0 |
| n\_weak\_mapping | 26957 |
| n\_ref\_term | 69 |
| n\_analysed\_pairs | 200000 |
| n\_accepted\_pairs | 200000 |
| n\_trans\_pairs | 96593 |
| n\_cis\_pairs | 103407 |
| n\_fully\_aligned | 249375 |
| n\_align\_term | 150625 |
| n\_no\_site\_end | 267149 |
| n\_short\_inserts | 22893 |
| classification | |  |  |  |  |  |  |  |  | | --- | --- | --- | --- | --- | --- | --- | --- | | informative | |  |  | | --- | --- | | fr | 26261 | | rf | 20508 | | ffrr | 42588 | | | uninformative | |  |  | | --- | --- | | religation | 3465 | | dangling\_ends | 13599 | | self\_circle | 116 | | ffrr | 335 | | |
| obs\_insert\_mean | 297.17234345057796 |
| obs\_insert\_median | 295.0 |
| mean\_readlen | 127.0 |
| unobs\_fraction | 0.1932203389830508 |
| digest\_stats | |  |  | | --- | --- | | cs\_start | 59371 | | cs\_term | 132851 | | cs\_full | 5430 | | read\_thru | 123326 | | is\_split | 87264 | | vestigial | - GATC - GANT | |
| separation\_bins | |  |  | | --- | --- | | bins | - 1000 - 5000 - 10000 | | counts | - 80514 - 63992 - 54619 | | vs\_all\_cis | - 77.86126664539151 - 61.8836248996683 - 52.819441623874596 | | vs\_accepted | - 40.257 - 31.996000000000002 - 27.309499999999996 | |
| digestion | |  |  |  |  |  |  |  |  |  |  |  |  |  |  |  |  |  |  | | --- | --- | --- | --- | --- | --- | --- | --- | --- | --- | --- | --- | --- | --- | --- | --- | --- | --- | | cutsites | |  |  |  |  |  |  |  |  | | --- | --- | --- | --- | --- | --- | --- | --- | | GATC | |  |  | | --- | --- | | name | DpnII | | site | GATC | | site\_len | 4 | | | GANTC | |  |  | | --- | --- | | name | HinfI | | site | GANTC | | site\_len | 5 | | | | junctions | |  |  |  |  |  |  |  |  |  |  |  |  |  |  |  |  |  |  | | --- | --- | --- | --- | --- | --- | --- | --- | --- | --- | --- | --- | --- | --- | --- | --- | --- | --- | | GATCGATC | |  |  | | --- | --- | | enz5p | DpnII | | enz3p | DpnII | | junction | GATCGATC | | vestigial | GATC | | junc\_len | 8 | | vest\_len | 4 | | pattern | GATCGATC | | cross | True | | | GATCANTC | |  |  | | --- | --- | | enz5p | DpnII | | enz3p | HinfI | | junction | GATCANTC | | vestigial | GATC | | junc\_len | 8 | | vest\_len | 4 | | pattern | GATCA[ACGT]TC | | cross | False | | | GANTGATC | |  |  | | --- | --- | | enz5p | HinfI | | enz3p | DpnII | | junction | GANTGATC | | vestigial | GANT | | junc\_len | 8 | | vest\_len | 4 | | pattern | GA[ACGT]TGATC | | cross | False | | | GANTANTC | |  |  | | --- | --- | | enz5p | HinfI | | enz3p | HinfI | | junction | GANTANTC | | vestigial | GANT | | junc\_len | 8 | | vest\_len | 4 | | pattern | GA[ACGT]TA[ACGT]TC | | cross | True | | | |
| junction\_frequency | |  |  | | --- | --- | | DpnII/DpnII GATCGATC | 60634 | | DpnII/HinfI GATCAATC | 7324 | | DpnII/HinfI GATCACTC | 6245 | | DpnII/HinfI GATCAGTC | 5039 | | DpnII/HinfI GATCATTC | 487 | | HinfI/DpnII GAATGATC | 784 | | HinfI/DpnII GACTGATC | 7510 | | HinfI/DpnII GAGTGATC | 9378 | | HinfI/DpnII GATTGATC | 11284 | | HinfI/HinfI GAATAATC | 144 | | HinfI/HinfI GACTAATC | 1415 | | HinfI/HinfI GAGTAATC | 1951 | | HinfI/HinfI GATTAATC | 2091 | | HinfI/HinfI GAATACTC | 173 | | HinfI/HinfI GACTACTC | 1300 | | HinfI/HinfI GAGTACTC | 1560 | | HinfI/HinfI GATTACTC | 1920 | | HinfI/HinfI GAATAGTC | 98 | | HinfI/HinfI GACTAGTC | 950 | | HinfI/HinfI GAGTAGTC | 1243 | | HinfI/HinfI GATTAGTC | 1400 | | HinfI/HinfI GAATATTC | 3 | | HinfI/HinfI GACTATTC | 104 | | HinfI/HinfI GAGTATTC | 164 | | HinfI/HinfI GATTATTC | 125 | |
| remnant\_frequency | |  |  | | --- | --- | | DpnII GATC | 83791 | | HinfI GAAT | 2364 | | HinfI GACT | 12514 | | HinfI GAGT | 15707 | | HinfI GATT | 18475 | |
