# Supplementary material for: qc3C: Reference-free quality control for Hi-C sequencing data
Source: PLoS Comput Biol. 2021 Oct 11;17(10):e1008839. doi: 10.1371/journal.pcbi.1008839 (PMC8530316; doi:10.1371/journal.pcbi.1008839)
Supplement: Attachment — Submitted filename: responses_to_reviewers.pdf [file pcbi.1008839.s006.pdf]

Responses to the Reviewers

The following text began as a copy of the “Comments to the Authors” we received.

Each of our (the authors) responses occurs immediately after the relevant reviewer comment. All author responses are in blue text and there is a small amount of unrendered LaTeX script when inline math was included in our responses.

To all:

After adding a new figure in response to feedback (fig10), we decided to remove black borders on all three similar 5-panel full-page figures. Although this has meant that the median is no longer visible in the boxes, we feel this has improved the clarity of low-dispersion bins where the legend colours were being completely obscured by the black borders. The font size has also been increased for these figures.

Data backing Figure 10 and the generating script have been added to our Zenodo archive. Consequently, the DOI within the manuscript has been updated.

Comments to the Authors

Reviewer #1:

This manuscript presents a new tool for evaluating the quality of Hi-C data, with or without a reference genome. The statistical method seems appropriate and based on reasonable assumptions, and there is currently no other method to satisfactorily assess the quality of a Hi-C library without a reference genome. While traditional sequencing QC measures can be used in the presence of a high-quality reference, qc3C presents an option for QC without a reference, which is likely to become more common as Hi-C is used more outside of humans and traditional model organisms. Some minor changes to improve clarity of the manuscript would be helpful, but otherwise the method is presented well and is likely to be useful to the community.

1. The figures overall are very high-quality and helpful, but it would be great if an extra figure could be added describing the qc3C method itself. It is a bit hard to follow the text alone in the methods section, and a visual aid here would be great. If there is concern over too many figures, I think Figure 1 could be removed (just cite the original Hi-C paper from 2009 which has a very similar figure to describe the protocol).

To aid the description of the qc3C algorithm, a workflow of the algorithm has been added and referenced in the text. This has been made supplementary so as to provide sufficient detail as the figure is quite large (figure S1).

2. The method requires an exact sequence match to identify a ligation artefact (LA), and results suggest that qc3C is more likely to underestimate the proportion of valid proximity ligation pairs. Would allowing for indels or sequencing error when counting LAs potentially improve this undercounting? Or is the exact sequence match likely to capture all LAs, and the underestimates are due to other factors?

The reviewer has touched on an important detail that we have spent time exploring in the development of qc3C. With our current analytical approach, in testing we found that weakening the exact match criteria for LA detection -- what is typically only an 8-mer -- was not clearly beneficial to prediction performance. The expansion of possible matches is further aggravated by experimental protocols (like Arima) which employ restriction enzymes with non-specific bases in their recognition sites.

Minor points for clarity:

1. My personal preference for ease of reading would be to discuss the results of the simulation and real data analysis right after the relevant data are described, rather than splitting these across sections so the reader has to flip back and forth to remember the experimental set-up when reading about the results.

We feel that this suggested alternative format would be a departure from the PLOS guidelines and therefore we would prefer explicit editorial guidance before making such a change

2. There are many acronyms introduced in the paper, and it becomes harder to remember them by the end. Especially for those used in figures, it would be helpful to put the full terms in the caption (ie, FF/RR/FR/RF in Figure 7 refer back to terms used in Figure 2, and are easy to have forgotten).

The Fig7 caption now reads:

"The results of qc3C bam mode analysis showing the breakdown of valid and invalid pairs for each library. Valid pair classes are shades of green, while invalid pair classes are shades of red, while two-letter acronyms refer to read-pair orientation (F: forward, R: reverse)."

3. For the real data results, it would be easier to follow if the libraries were renamed to something descriptive/interpretable, with a table mapping these to the official library names (or just add a column with these descriptive names to Table 1). It is very difficult to interpret Figure 8 without flipping through the text multiple times to find the descriptions of each sample to determine which are human and which are turtle, and what the differences are between them all. For example, DRR177157 could be referred to as "P.sinensis_HindIII" both in the text and in the figure, so the reader automatically knows the species and defining characteristics of this library.

Working names which encode some of the run details have replaced the accession IDs. This has meant modifying the tables, text and figures to suit.

Reviewer #2:

In this manuscript, authors present a new tool, qc3C, which is able to control the quality of Hi-C libraries without the need of an assembled genome by using the uneven Hi-C libraries k-mers distribution generated by ligation artifacts. This QC toolbox will be of particular interest to researchers wanting to assemble new genomes, or especially metagenomes, using Hi-C libraries. It is also an interesting tool for any Hi-C as an "all-in-one" Hi-C QC toolbox, providing enzyme configurations for Arima and Phase Hi-C kits, which makes it easy to use.

The manuscript is well written, the figures are clean and the tool will be of use to the field. The provided Zenodo repository was greatly appreciated during the review process, as well as the manuscript formatting. Despite the evident quality of the manuscript, major key points are lacking for the manuscript to be readily accepted for publication.

- The proper figures and analyses to clearly show how the reference-free mode compares to the more precise bam mode to validate the quality of Hi-C libraries. Table 2 is insufficient to that end. For instance, scatter plots displaying "read thru" bam compared to no-reference Hi-C reads fraction, and bam mode valid pairs compared to no-reference Hi-C fraction would help to understand how these different metrics are related to one another for valid and low quality Hi-C libraries.

We have added a new figure (figure 10) which compares our reference-free approach to HiExplorer's hcQC report via the simulated sweep. This includes additional discussion to accompany it. We think this helps to highlight pros/cons of these different approaches while introducing the least amount of complication.

Please refer to comments below regarding HiCExplorer and Table 2 for greater detail about this additional work.

- A table showing the currently available Hi-C QC tools with their respective features would help highlighting the qc3C features and what it adds to the field.

Other software has been mentioned in the body of the manuscript and we feel that it is clear that the key feature offered by qc3C is the reference-free estimation of the Hi-C fraction. Further, we make the claim that this is the first example of such functionality. The manuscript goes into detail as to why this is important for certain fields in genomics and the general logistical advantages.

- Figure 7 should compare results obtained by qc3C bam mode and Hi-C Pro QC tool for each type of read pairs. Comparing qc3C bam mode to HiCExplorer hicQC is also important to assess how qc3C performs compared to other tools.

In these first three points, the authors are concerned that reviewer #2 is overlooking that the primary significance/novelty of qc3C is the ability to infer the Hi-C fraction without a reference. Adding these recommendations would lead to an over-emphasis on reference-based statistics, which were not intended to match 1-to-1 existing tools, for which these statistics were not their primary concern.

- Figure 8 should be changed to show interactions larger than 1Mb range. Biologically relevant interactions are seen at that range. Moreover, monitoring contacts above the 1Mb range between Hi-C libraries help to determine how comparable Hi-C datasets are.

The primary purpose of qc3C is to infer the fraction of Hi-C reads and figure 8 is intended as a demonstration of part of the MultiQC visual report available. Further, qc3C does not currently compute counts of pair separation greater than 1Mbp and therefore this figure cannot be amended and remains a true demonstration of qc3C's current capability.

We have added this as a feature request to our repository (issue #54), although we have no control over the timing of official releases of MultiQC.

- I was unable to test the tool on a computing cluster. After following the installation process detailed on the github page of the tool, using the bam mode, I received an error concerning “/lib64/libm.so.6: version `GLIBC_2.23' not found”. After installing the tool on my local machine I was able to test it and it seemed to work in bam mode. [insert new line]

We are aware that older Linux distributions (roughly pre-2016) may experience this issue when using our basic Conda installation and we have added additional specific notes to qc3C’s documentation to assist them. We would like to point out that this issue is not limited to qc3C, affecting Conda packages built on Linux distributions released in the last few years and whose dependencies eventually reach glibc (PyTorch and Tensorflow to name two well known projects).

For qc3C, users have two documented ways to resolve this:

1. Take advantage of our Docker (Singularity compatible) image.
2. Use our described two-step (two command) procedure.
    a. Create a conda environment with Jellyfish and Python
    b. Install qc3C using Pip from Github

The second option allows distributions to use their system’s existing installation of glibc.

[continued] However I was unable to load separated bam files produced by BWA for HiCExplorer (R1.bam and R2.bam, i.e. non-merged bams). Such a mode would be necessary so that qc3C can be used with any type of Hi-C bam file (splitted or merged).

The feature request for split BAM files has been added to our Github repository (issue #51).

- The methodology concerning the reference-free mode, while explained rather thoroughly, requires a supporting figure explaining each step so that the method can be better understood. For instance, authors could provide a figure explaining the two methods parts (observed fraction and correction for the unobserved).

A workflow of the algorithm has been added and referenced in the text. This has been made supplementary as to provide sufficient detail, the figure is quite large.

- A figure explaining the inputs and outputs and connexion between the three CLI tools of qc3C should be provided.

A new figure has been created which visually represents the input and outputs, which we will include as a supplementary figure and add to our GIthub repository.

- An example of the outputs of the tools should be provided in the article in a complete figure.

For BAM and KMER modes, examples of the JSON and HTML output will be included as supplementary files and added to our Github repository. The library creation mode does not produce a report, while an example database seems of less value to the reader.

We have added "example data and results" as a feature request on our Github repository (issue #49).

There are also Minor comments to address:

- qc3C should provide the number of duplicated sequences in the multiQC report.

This has been added as issue on our Github repository (issue #50). The speed at which this change will carry through to an official release of multiQC is out of the authors control.

- Figure 2 is cited in the text before Figure 1

Fixed

- Figure 2 has several issues that need to be corrected. Change Figure 2 Top and Bottom to Figure 2 A and B. Figure 2 Invalid pairs figure is missing some cases of pairs that should be removed from Hi-C datasets, for instance singletons / one mate unmapped reads. Check for instance Lajoie et al. 2015, “The Hitchhiker's Guide to Hi-C Analysis: Practical guidelines” Figure 2, or the HiCExplorer hicQC documentation (https://hicexplorer.readthedocs.io/en/latest/content/tools/hicQC.html). The “religation” invalid pairs on Figure 2 bottom schematic seems to be a valid pair from most common representations of Hi-C reads.

The references to "top" and "bottom" have been changed to A and B and the text has been revised.

For the sake of brevity, the figure has been limited to cases in which both ends map to the reference, as these situations deserve more explanation than pairs which fail to meet this basic criterion. That said, we have now added an explicit note to the caption rather than extend the figure further.

This note reads:

> "Further invalid cases exist which are not shown, these include cases of unpaired reads and when one or both ends of a pair do not map to the reference."

Regarding "religation", although these pairs are a product of the steps of digestion and ligation, their production is not desirable. This is because, in the context of what information can already be obtained efficiently through conventional shotgun sequencing, they fail to provide new information. It could be argued that a more apt set of terms might be informative/uninformative rather than valid/invalid, but we chose not to digress into this discussion. The requirement of non-adjacency -- where at least two cut-sites must occur between reads -- places religation squarely in the invalid category. In saying this, we recognise that the definition is not entirely consistent throughout the Hi-C literature.

- Long range pairs are much farther than 1000bp, especially since 1kb resolution matrices are common in Hi-C and 1000bp represents two adjacent bins, i.e. the closest possible range available in most Hi-C experiments. Long range contacts are depending on the chromosome scales and genomes. HiCExplorer for instance chooses to separate short and “long” range contacts at the 20kb threshold. But interesting and biologically relevant interactions are seen above the 1Mb range in many organisms. That 1000bp threshold must be changed in the text or specified that it concerns genomes with very small chromosomes. Also, it is advised to think in terms of gradual genomic separation and ranges rather than the binary short vs long range interactions - that is precisely what Distance vs Counts corresponds to (number of contacts in Y, log10 genomic separation in X, represented in Figure 8) and it is a much better QC assessment than proportion of long range vs short range interactions.

The authors wholeheartedly agree that long-range Hi-C pairs, which researchers hope are captured in quantity and subsequently leveraged in downstream analyses, should be smoothly distributed at distances much greater than 1kbp.

However, the simple 1kbp, 5kbp, and 10kbp statistics are not intended to consider this aspect but rather are a simple parsimonious test of whether a Hi-C library is producing pairs which map with separations greater than can be plausibly explained by the sequencing of conventional inserts. That is to say, an insert which produces a pair which map at distances

much greater than the mean insert length of the library (perhaps this threshold begins at 1kbp) is likely to be a product of proximity ligation. Of course, there are other possible mechanisms that contribute to a FPR of this simple pair separation statistic.

From our investigations, the mean insert size of published Hi-C libraries frequently falls below 300bp. Treated as normally distributed, a statistically conservative rejection threshold would be the mean (μ) + 2.5-3x stddev (σ).

For simplicity -- and noting the historical tendency in genomics for this -- a round figure of 1kbp was chosen as the smallest threshold.

Lastly, we have added “>20kbp” as an enhancement (issue #57) to our Github repository.

- Authors need to describe more in-depth what is the expected frequency of reads displaying the sequenced ligation junctions (line 150). Dangling ends should most likely display the ligation junction sequence while most valid pairs shouldn’t display the ligation junction (depending on the library fragment size and reads length).

We are unsure what the reviewer is referring to here regarding a request for more depth.

We note: During its production, a dangling-end involves only one digested recognition site. Consequently, this will not lead to the duplication of two digested overhangs aka the ligation artefact.

- Each part of “Observed fraction” should be splitted in sub parts with subtitles (k-mer extraction, bootstrap...) to improve the comprehension of the method. It would also support the required new associated figure (Method section starting line 154).

As suggested, this subsection has now been split into four with the titles:

- Construct k-mer database
- Extract local k-mers
- Calculate relative frequency
- Bootstrap confidence interval.

- How n = 16 has been determined is unclear (line 159).

A thorough analysis of the response of our reference-free estimation to the value of n has not been provided as we have found it to be reasonably insensitive over a practical range (16 <= *n* <= 24) and the variation lacks a clear trend of performance improvement or

degradation over simulation sweeps. We expect this is due to the limits of fidelity of the Hi-C data simulator -- which is the basis of the ground-truth.

The default value of 16 was instead chosen on the pragmatic terms mentioned in the manuscript, page 6 lines 156-160.

Since the variable *n* was only introduced for the purpose of description, we have elected to remove it and only refer to *k*. We have also added additional discussion within this section for clarity.

The “Construct *k*-mer database” subsection has been revised as follows:

> “To query the frequency of arbitrary \textit{k}-mers, k is first chosen and a frequency database is built from the supplied Hi-C readset using the \textit{k}-mer counter Jellyfish \cite{Marcais2012-bc}.
>
> As we intend to query the frequency of local \textit{k}-mers flanking a LA identified within a read, for the sake of specificity, it is necessary that k be substantially larger than l_{LA}, where l_{LA} is the LA length. For modern Hi-C protocols in common use $l_{LA}=8$, while for the older 6-cutter protocol based on HindIII $l_{LA}=10$. The size of k, however, also defines the total width of the surrounding region and limits how close we can approach either end of a read and still carry out our local \textit{k}-mer frequency inspection. A trade-off, therefore, exists between increased specificity of local \textit{k}-mers and decreased sensitivity due to the resulting unobserverable region of each read. In testing, we have found values between $24 \le k \le 32$, to be similar in their predictive performance. A default of $k=24$ strikes a balance between these two competing processes, while also limiting database file size and query performance on small-memory machines.”

- The size of the random set of paired reads isn’t defined (line 160).

The size of this set is user-defined.We expect that users will choose a sample size that suits their needs.

- How many bootstraps are performed on the table T of LA presence/absence per read to assess the confidence interval (line 178)?

This is a user controllable parameter, with a default of 100. The text has been amended to mention the default as follows:

> “Lastly, from the total pool of sample estimates ${\rho_n}$ (default: 100 bootstrap samples) ...”

- “Reduced to a unique set of values” is not clear (line 182).

We have attempted to make this clearer.

> "From this sample, the collection of relative frequencies ϕ_i for observations which did not contain the LA ($\lambda_i = 0$) are selected and reduced to a unique set of values (duplicate observations removed)."

- The origin of the p-values is not clear (line 183).

A mathematical definition and practical citation covering empirical p-value calculation has been added.

- On which data the bootstrap is performed is not clear.

The bootstrapping is performed on the table of observations T_n. We have reworded this subsection's initial sentences.

> "Following the above procedure and iterating over a set of reads, a table of relative frequency observations is constructed ($T = {(\phi_i, \lambda_i)}$), where λ_i is a binary indicator variable for the presence/absence of the LA sequence ($\mathrm{present} \rightarrow \lambda_i=1$).
>
> Bootstrap sampling of this table permits a confidence interval to be estimated for the fraction of observed LAs which were produced through the proximity ligation reaction (true LAs) as follows. ..."

- Are Merged and unmerged read pairs (line 209-210) corresponding to overlapping and non-overlapping read pairs sequenced from the same fragment (line 200)? The nomenclature should be the same in the whole manuscript.

We thank review #2 for pointing out this missing detail. An additional paragraph has been added to this subsection, as follows:

> "Alternatively, pre-processing can be applied to pairs with sufficient overlap; merging them into a single sequence. As a consequence, the complete insert sequence of such merged pairs can be easily inspected and the possibility of duplicate observations eliminated. However, since a library is unlikely to be composed entirely "merge-able"

> read-pairs, pre-processed libraries require the simultaneous handling both merged and unmerged pairs."

- What are generated pairs corresponding to (line 229)? The number of simulated Hi-C reads?

We feel this is clear to the reader as the paragraph in which this issue has been raised begins by introducing the simulation's experimental design using the language ".. *where reads were generated using the Hi-C read simulator sim3C ...*"

- Authors should compare the results of the qc3C reference-free mode, qc3C reference mode, and already published Hi-C QC tools (most likely reference-based) like HiCExplorer hicQC module so that the readers can assess how precise qc3C reference-free and reference-based reports are compared to the tools that already published.

We would like to point out that **Table 2** already presents a quantitative comparison between qc3C's reference-free and referenced-based results and there is accompanying discussion beginning at line 326.

The discussion points out that the fraction of Hi-C pairs inferred from our reference-free method does not perfectly align with the conventional "number of hi-c contacts" statistic. This is because the latter is the end product of many simple criteria, whose collective aim is to produce a min-noise/max-signal contact matrix. In contrast, qc3C's reference-free method attempts to estimate the entire fraction of Hi-C pairs, regardless of the separation distance for any individual pair or whether it would provide useful contact information.

As such, we expect that reference-based counts will under-estimate the true fraction. And indeed, we observe this in testing (see below).

Even so, we have now calculated HiCExplorer QC statistics for the entire simulated sweep, and added a new figure 10. The accompanying discussion is as follows:

> "For comparison, the tool hicQC (default options) from the HiCExplorer suite [18,19] was used to infer signal content. Here, signal content was calculated as the ratio of the reported statistic "Hi-C contacts" against the number of reads analyzed. When the sweep is partitioned in equivalence to our reference-free method above (figure 10), it can be seen that signal content was underestimated for most simulated scenarios, decreasing steadily -- for the most part -- as insert size increases. The exception to this trend occurs for small insert sizes (150 -- 200 bp) and lower signal levels (0.01-0.25), at which point overestimation can occur. In particular, when insert size was equal to read length (150 bp) and signal content was lowest (0.01), signal content was more than

20-fold overestimated. Variation between simulation replicates follows typical counting statistics, decreasing steadily as signal content increases.

Given sufficient coverage ($\geq$ 3.2-fold), our reference-free estimation method compares favourably with the quality metric derived from HiCExplorer quality statistics, obtaining as good or better estimations of signal content. However, it must be remembered that the "Hi-C contacts" statistic reported by hicQC is equivalent to the number of pairs accepted by HiCExplorer when constructing the contact matrix for a given library. As such, used as a measure of signal content, this value should be expected to under-report -- relative to the true value -- as each pair must pass multiple criteria intended to minimise the false-positive rate (noise) within the matrix."

- qc3c --help should display what each available commands (mkdb, bam and kmer) are doing.

Each mode does go into detail, but we agree it would be helpful if more information was available at the top level (qc3C --help). We have added this to our Github repository (issue #53)

- qc3c bam mode: -q MIN_MAPQ, --min-mapq MIN_MAPQ. Minimum acceptable mapping quality [60]. The default value of 60 is potentially dangerous as a default parameter depending on the mapping tool used to generate the BAM file.

This has been made an issue on our Github repository. It will require testing with other mapping tools before we can mark it resolved (issue #52).

- qc3c bam mode: -b parameter documentation is not clear about the possibility to use multiple bam files.

An issue regarding split bam files has been created on our Github repository and this detail has been included (issue #51).

- qc3c bam mode: the check for bam file index should be performed before any computation.

Checking for the existence of an index is not necessary. For qc3C, BAM files must be query-name sorted and are consequently parsed in sequential order making an random-access index of limited value. Further, qc3C does check for the correct sorting.

Perhaps this comment is motivated by a warning emitted by recent releases of Pysam. We have not yet thoroughly investigated the cause within the Pysam and HTS lib codebases and so have not suppressed it. Its appearance has not marked any change in qc3C's behaviour or output.
